# Efficient Proximity Computation Techniques Using ZIP Code Data for Smart Cities †

**DOI:** 10.3390/s18040965

**Published:** 2018-03-24

**Authors:** Muhammad Harist Murdani, Joonho Kwon, Yoon-Ho Choi, Bonghee Hong

**Affiliations:** School of Computer Science and Engineering, Pusan National University, Busan 46241, Korea; hariste@gmail.com (M.H.M.); yhchoi@pusan.ac.kr (Y.-H.C.); bhhong@pusan.ac.kr (B.H.)

**Keywords:** proximity computation, data models, ZIP code data, smart city

## Abstract

In this paper, we are interested in computing ZIP code proximity from two perspectives, proximity between two ZIP codes (*Ad-Hoc*) and neighborhood proximity (*Top-K*). Such a computation can be used for ZIP code-based target marketing as one of the smart city applications. A naïve approach to this computation is the usage of the distance between ZIP codes. We redefine a distance metric combining the centroid distance with the intersecting road network between ZIP codes by using a weighted sum method. Furthermore, we prove that the results of our combined approach conform to the characteristics of distance measurement. We have proposed a general and heuristic approach for computing *Ad-Hoc* proximity, while for computing *Top-K* proximity, we have proposed a general approach only. Our experimental results indicate that our approaches are verifiable and effective in reducing the execution time and search space.

## 1. Introduction

Proximity is a measure of closeness between two or more correlated objects. It is used for finding a nearby hotel, target marketing, disease outbreak analysis, social network analysis [1], and identification of a false insurance claim. An efficient proximity computation can be one of the crucial services for smart cities [2]. Different application domains use different measurements, but the service requirements are always the same.

- The measurement used for computing proximity should be valid and justifiable. The validity can be explained by proving that the measurement is a distance measurement. A justifiable measurement is one that has a strong logical reason and an effect on the increasing or decreasing proximity.
- The proximity computation system should be able to solve at least two basic proximity computation problems: computation of the proximity between pairs of objects and that of neighborhood proximity, which are called *Ad-Hoc* and *Top-K* proximity, respectively.

**Figure 1:**
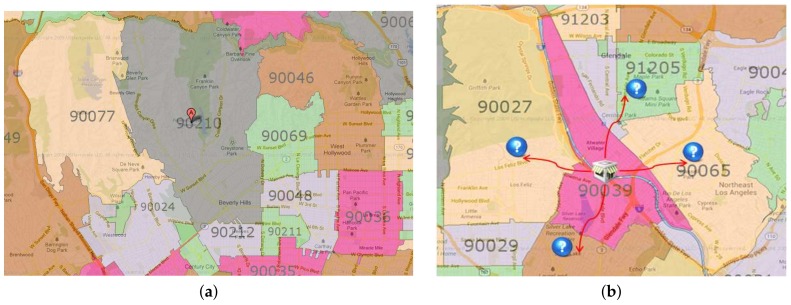
ZIP code data and their application. (**a**) ZIP code information; (**b**) targeted marketing example.

The ZIP code boundary dataset contains spatial data that represent the divided areas within the United States. ZIP codes can be helpful for finding or traveling between two designated locations more efficiently and quickly [3]. Figure 1a shows part of the adjacent ZIP code with 90210 as the center.

ZIP code proximity can be used as a primary tool for targeted marketing in a business such as supermarket chains. A grocery store generally tries to gather the consumer location data with ZIP code data. A store owner can easily find a suitable area for a targeted campaign or advertisement by computing ZIP code proximities. Figure 1b shows an example case of ZIP code proximities in which a store is located in 90039. If the owner of the store knows the ZIP code proximities in a sorted order, he can decide to focus on the Top-K ZIP codes for targeted marketing. This decision will reduce the marketing cost very effectively.

Several systems [4,5,6,7] focus on solving the issues of computing proximities. Threshold algorithms for graphs (TAGs) support proximity queries around a source node (neighborhood proximity) rather than on proximity computation between node pairs. A random walk with restart (RWR) [5] technique utilizes the steady-state probability for providing proximity (relevance) scores between two nodes in a weighted graph. A top-k spatial keyword query system [6] allows users to manage the preference weights and individual keyword in an intuition-consistent way. A top-k spatio-textual preference query [7] considers not only the spatial location, but also additional information such as ratings. Nonetheless, these systems do not exploit the ZIP code and variant weights of road types during the proximity computation.

Our motivation for this work is to enhance the correctness of the ZIP code proximity computation. This is a challenging problem. The issue here is that, if we are using Euclidean distance as the sole measurement, we will obtain a delusional result because a real landmark is not always planar and plain. There will be a hindrance or barrier such as mountains, hills and cliffs. Thus, we need to formulate a new definition for distance and find other measurement to support the distance correctness.

To further address these challenging issues, we designed and implemented a system to support efficient proximity computation techniques for ZIP code graph data. In the preprocessing step, we transform the ZIP code spatial data into a ZIP code graph. To compute the proximity, we formally define and combine two proximity measures, which are the adjacent ZIP code distance and the weight of the intersecting roads. The more common boundaries do not really increase the proximity, but the greater the number of intersecting roads, the higher the proximity. We assume that only the road network is being used and do not consider other means of transportation such as ferries, trains, or airplanes.

To justify the correctness of our proximity measurement, we provide the mathematical proof and experiment test case that employs a different set of ZIP codes. Conforming to the service requirements, we propose efficient proximity computation techniques for non-adjacent ZIP codes in the case of *Ad-Hoc* and *Top-K* proximity computation.

The key contributions of this paper are summarized as follows:

- **A new distance measures for ZIP codes**: Aside from the centroid distance, we introduce a new distance measurement for proximity called intersecting road weight between adjacent ZIP codes. We assign different weights on the basis of the road types, namely primary road, secondary road, and other road. We combine the intersecting road weight with the centroid distance for computing the proximity in a graph of ZIP codes. The weighted sum is used for combining and preserving the distance metric properties.
- **Proximity measures validity**: We prove that our combined proximity measures conform to the special characteristic of distance measurement. Thus, we can say for sure that the greater the distance between two objects, the smaller is the proximity.
- **Efficient proximity computation techniques**: We proposed an algorithm to efficiently compute the proximity function using the centroid distance and the intersecting road weight by minimizing the search space.
- **Experimental evaluation**: Last but not least, we justify the correctness of our proximity measurement and evaluate the performance of *Ad-Hoc* and *Top-K* proximity processing using a ZIP codes graph. We construct the ZIP codes graph on the basis of a real ZIP codes data set.

The rest of this paper is organized as follows: Section 2 introduces the related work. Problem definition and proposed system architecture are explained in Section 3. The details of the proposed approach, which includes graph data modeling, graph pre-processing, proximity equation, and computation are explained in Section 4 and Section 5. While the experimental result is presented in Section 6. Finally, we present our conclusions in Section 7.

## 2. Related Works

In this section, we will review the existing works on proximity computation in a graph and divide it into two subsections, proximity in a graph and minimum weight computation algorithm. The graph here is undirected and either unweighted or weighted with a non-negative value. We will also use the term *node* and *vertex*, which has the same meaning and can be used interchangeably.

### 2.1. Proximity in a Graph

Distance is the common measurement for computing proximity. The definition of distance mostly depends on the graph data domain. Euclidean distance is used in the road network and other spatial data-based graph. For an unweighted graph, distance is defined as the number of nodes that need to be traversed on a path for connecting two nodes.

In a graph of a social network, the use of proximity falls into two categories: person search [8] and link prediction [9,10,11]. Since links tend to exist between close nodes, the simple neighborhood-based measures use the common neighbor’s number. If *s* and *t* have a high number of common neighbors, it means that they are likely to be closely related to each other.

A random walk with restart (RWR) technique [5] measures the proximity between source *s* and target *t*, and they perform a random walk. A proximity value is determined using the steady-state probability. TAGs [4] use the product of the shortest path distance and the maximal network flow to compute the proximity. Originally, maximal network flow is not a distance measure but can be converted into one by subtracting the maximum flow value. However, the method focuses on calculating only the neighborhood proximity, similar to top-k proximity. In contrast with TAGs, our proposed approach calculates both node pairs and neighborhood proximity.

Table 1 provides a brief summary of research on proximity computation in a graph.

### 2.2. Top-K Spatial Queries

Efficient processing of top-k queries is a crucial operation in many applications that involve huge volumes of data. Marian et al. suggested sequential strategies for evaluating top-k queries over web accessible databases [12]. A comprehensive study of top-k query processing in relational databases is found in [13].

Due to the popularity of location-based services, retrieving top-k spatial queries has gained increasing attention recently [6,7,14,15,16,17,18]. A comprehensive experimental evaluation of different spatial keyword query indexing and query processing techniques has been surveyed in [15]. Yiu et al. computed the score of a data object based on feature objects in its spatial neighborhood from multiple feature sets [14]. Tsatsanifos et al. proposed a top-k spatio-textual preference queries similar to our work [7]. Zheng et al. proposed an interactive top-k spatial query [6] that can learn the users’ preferences automatically based on their feedback. Cho [16] proposed an efficient algorithm for top-k spatial preference search (ALPS) that grouped the data objects in a road segment, transformed them into a data segment, and generated a skyline set for each data segment [16]. A collaborative approach to moving k nearest neighbor (COMET) focused on continuously finding the k nearest neighbors (NNs) of a moving query object in directed and dynamic road networks [17]. Recently, Luo et al. investigated reverse spatial and textual k nearest neighbor (RSTkNN) queries on road networks [18]. However, none of the previous work considers using the ZIP code and different weights of road types during the proximity computation.

Our initial work [19] only explains the preprocessing and proximity equations using the centroid distance and common boundaries and did not provide the experimental result. However, the use of the common boundary point for computing proximity has a fatal flaw. First, it is ambiguous as sometimes few points have a relatively long boundary length. We should focus on measuring the common boundary length, not on counting the number of points. Second, even if we focus on the boundary length, not all of it can be classified as a road. Depending on the landmark type, these long common boundaries can be a desert, hills, or wasted area. Thus, we cannot really rely on the common boundary length for computing proximity. In this paper, we will use the intersecting roads between adjacent ZIP codes, extracted from TIGER/Line^®^ Roads spatial data [20], as one of the proximity measurement methods.

Table 2 describes a brief comparison of research on Top-K Spatial Queries.

### 2.3. Minimum Weight Computation Algorithm

The basis for computing the distance between two nodes in a graph is the summation of the minimum distance in a path. There are classic and well-known algorithms for computing the minimum distance. The algorithm can be differentiated by the existence of constraints or weights and estimate the distance to the destination node.

Breadth-First Search (BFS) is a classic algorithm that can be implemented with a queue for an unweighted graph [21]. For each adjacent node, BFS traverses to the adjacent nodes that were not visited yet. BFS computes the shortest path in O=(E+V) time. If the graph has special constraints such as edge weights, Dijkstra [22] is one of the most well-known algorithms for a weighted graph with non-negative values. The original Dijkstra does not use a priority queue for storing the set of unfinished vertices and runs in $O=(V^{2})$ time. Dijkstra with a priority queue implementing Fibonacci Heap runs in O=(E+VlogV) time. Based on the graph data domain, if we can estimate the distance to the destination, we can use A* search [23] (also known as heuristic search) to guide vertex selection in a greedy manner. Dijkstra can be considered an A* search with a heuristic value of zero. It achieves better performance than Dijkstra because of the minimization of search space.

Our naive approach for computing proximity based on the same concept as Dijkstra. It traverses through the neighborhood until it finds the target. The proposed efficient proximity computation is based on a heuristic estimate of distance. It is similar to the A* heuristic choice, past path, and future path, but we increase the degree of relaxation as we also consider the intersecting roads weight in the proximity computation. Table 3 shows comparison between research on the minimum weigh computation.

## 3. Problem Definition and Proposed System Architecture

In this section, we will define our problem and propose a solution. Section 3.1 explains the definition of ZIP code proximity computation and the emerging challenges in detail. Section 3.2 discusses the proposed system architecture, which consists of three main parts, namely a graph preprocessing module and a proximity engine, and a graph database storage.

### 3.1. Problem Definition

Supposing that z(s,t) is an *Ad-Hoc* pair that consists of *s*: a source ZIP code, and *t*: a target ZIP code; topk(s,k) is a *Top-K* pair, where *k* is the number of neighborhood nodes that we want to retrieve. We formally define the ZIP code proximity computation as follows:

Given a pair of z(s,t) or topk(s,k) and a ZIP codes graph G. Find the proximity value between s and t or find the neighborhood proximity of s that resulted in a rank of k and its proximity value from G by using the correct measurement.

**Figure 2:**
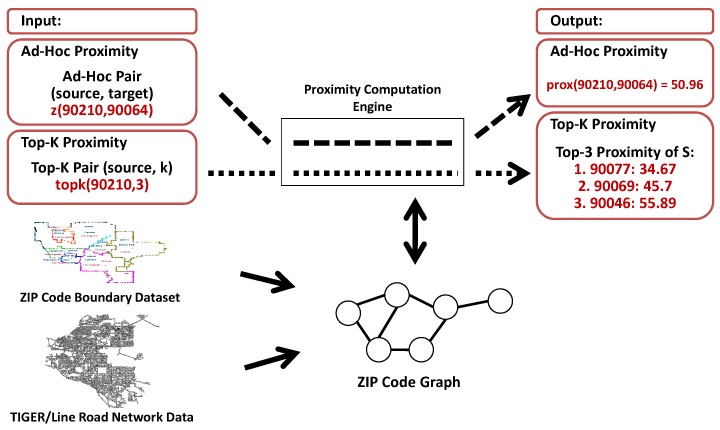
Visualization of problem definition.

Figure 2 shows a visualization of our problem. A ZIP code graph is built on the basis of a combination of the ZIP code boundary dataset [24] and the TIGER/Line road network data [20]. As shown in the figure, we can process the *Ad-Hoc* and *Top-K* proximity using the proposed system.

As mentioned before, there are several challenges of ZIP code proximity computation: (1) finding the right measurement for the proximity, (2) combining the measurements, and (3) finding efficient ways for solving proximity queries.

The first challenge is to find the right measurement for the proximity value. Assuming that we have the latitude and longitude information, we can calculate the corresponding direct centroid distance of it. As proximity is inversely related to distance, the closer the two points are, the higher the proximity value is. However, a real environment is not always as plain as planar drawing. Sometimes, when there is an impassable natural barrier that separates two locations, we need to take a detour to go from one place to another. The closer centroid distance may seem small, but, in reality, a considerable distance exists between them.

The second challenge is, if the several measurements are carried out more than once, how can we combine the various runs to compute the ZIP code proximity value? Which operator should we should use? Should we use subtraction, multiplication, or another method?

The last challenge is to find efficient ways to process the *Ad-Hoc* and *Top-K* proximity computation.

### 3.2. Proposed System Architecture

**Figure 3:**
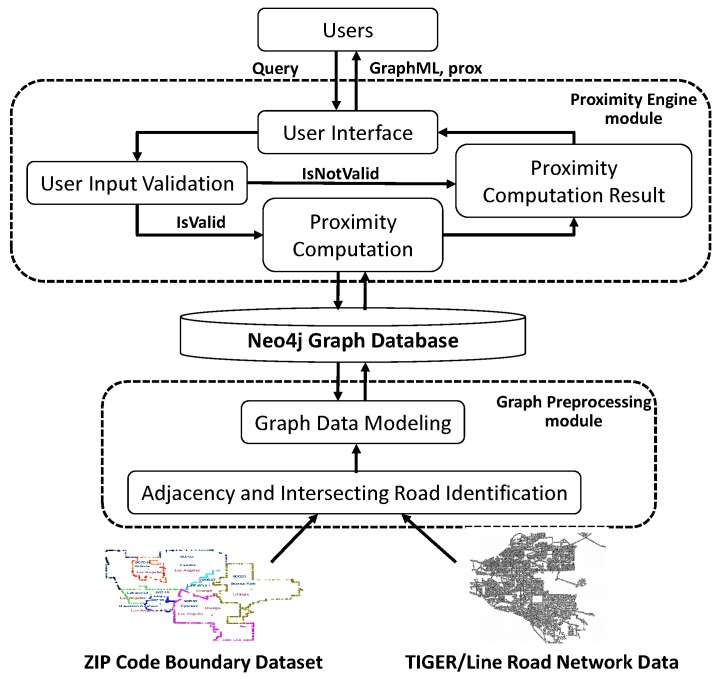
System architecture.

Figure 3 shows the proposed system architecture with two main modules and one graph database storage. To be able to compute the ZIP code proximity, we need to transform and combine the ZIP code boundary dataset and the TIGER/Line road network data into an undirected, weighted graph *G*. This is the function of the graph preprocessing module that uses the graph database storage. Then, the proximity engine module computes the ZIP code proximity by using the graphs.

The graph preprocessing module, which will be explained in Section 4, consists of two submodules: (1) adjacency and intersecting road identification, and (2) graph data modeling. As the name implies, the first submodule is used for identifying ZIP code adjacency from the ZIP code boundary dataset and intersecting roads from TIGER/Line road network data. The second submodule is in charge of combining the adjacency with the intersecting road and inserting it into the graph database storage.

The proximity engine module, which will be explained in Section 5, consists of four submodules: (1) user interface, (2) user input validation, (3) proximity computation, and (4) proximity computation results. The user interface submodule is used as a proxy to communicate with the user, either by receiving an input or sending the output results. The input will be validated by the second submodule. If it is valid, then the proximity computation submodule will run the process. If it is not, then the returned result will be invalid user input. Finally, the last submodule collects the computational results, builds an appropriate graph in GraphML file format, and sends it back to the user through the user interface submodule.

## 4. Graph of ZIP Codes

This section discusses graph data modeling, particularly the node and the edge type for our graph, and the data preprocessing shows the transformation of the raw data into graph data. All the work discussed in this section is covered by the graph construction module. Hereafter, the term of “raw data” refers to both the ZIP code boundary dataset and the TIGER/Line road network, unless specified otherwise.

### 4.1. Graph Data Modeling

In this subsection, we formulate a definition based on the characteristics of the raw data and then utilize the definition to model the required node and edge type. Because of the different requirements for data transformation and ZIP code proximity processing, we divide our graph data modeling into two parts: preprocessing and proximity computation. Preprocessing is concerned about the modeling, required node and edge type, during the raw data transformation. Based on the preprocessing result, we retain only a small number of nodes and edge types that are crucial for computing ZIP code proximity and remove the others.

**Figure 4:**
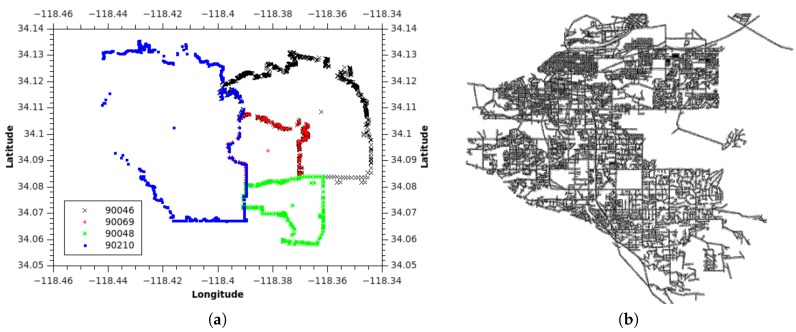
Raw data visualization. (**a**) ZIP code boundary; (**b**) TIGER/Line road network.

The ZIP code boundary dataset contains the ZIP code boundary points and a centroid (as shown in Figure 4a), while the TIGER/Line road network contains multiline data and the road properties (as shown in Figure 4b). Using the boundary points, we can extract the adjacency connection between ZIP codes. On the basis of the adjacency, we compute the centroid distance and identify the intersecting road. As each road has road type properties, we assign the roads’ different weight values.

**Definition** **1.** Common boundary length is the length of shared boundaries between two ZIP codes. It is used for defining the adjacency between them.

**Definition** **2.** Centroid distance is the Euclidean centroid distance between two adjacent ZIP codes. The larger the centroid distance, the lesser the proximity is.

**Definition** **3.** Intersecting roads are all of the roads that connect two adjacent ZIP codes. There are three types of roads: primary road (S1100), secondary road (S1200), and others (S1400). We assign a different weight to each road type. The larger the summation of intersecting roads weight, the higher the proximity is.

The nodes and edge types used for the preprocessing part are as follows: the node types are (1) *Polygon Node*, which represents one or more polygons for a ZIP code. Its properties are polygon id, zip id, and type; and (2) *Point Node*, which represents the boundary point of a polygon. Its properties are latitude, longitude and type. The edge types are (1) *IsInZip Edge* connects *Polygon* to *ZIP code*. Its only property is type; (2) *IsBounding Edge* connects *Point* to *Polygon*. Its only property is type; (3) *IsPolyAdjacent Edge* connects *Polygon* to another adjacent *Polygon*. Its properties are common boundary length and type; and (4) *IsConnectedTo Edges* connects *Point* to another *Point* in the same boundary as that of polygon. Its properties are point distance and type.

**Figure 5:**
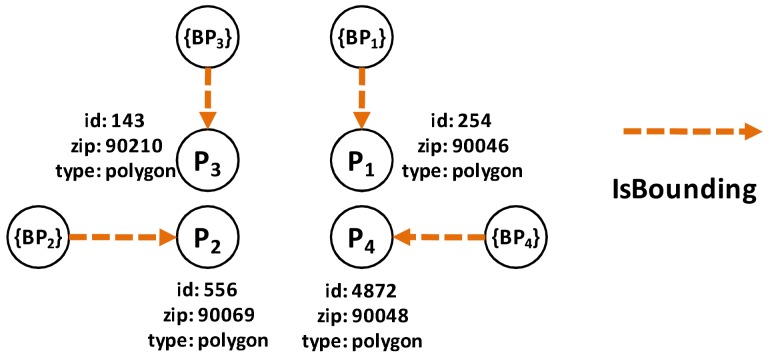
Boundary point and polygon node.

**Example** **1.** Let us use the example of the ZIP code boundary point shown in Figure 4. There are four ZIP codes in the data, namely 90046, 90069, 90048, and 90210. Each ZIP code in the example has exactly one polygon that is differentiated on the basis of color. Each polygon consists of multiple boundary points. Figure 5 depicts the boundary points and polygon node of the four ZIP codes. $P_{1}$ is the polygon node for 90046, and $\left\{BP_{1}\right\}$ is the set of boundary points of 90046. $P_{2}$ and $\left\{BP_{2}\right\}$ belong to 90069. For 90210, we use $P_{3}$ and $\left\{BP_{3}\right\}$, whereas $P_{4}$ and $\left\{BP_{4}\right\}$ are used for 90048.

**Figure 6:**
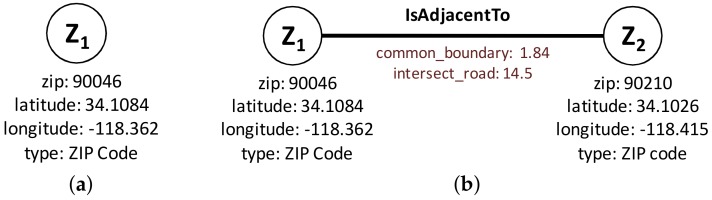
Graph data modeling-proximity computation. (**a**) ZIP code node; (**b**) IsAdjacentTo Edge.

Using the graph modeling results of preprocessing (IsPolyAdjacent Edge), we derive the ZIP code adjacency. Each polygon refers to a ZIP code, and a ZIP code may have one or more polygons. Thus, two main components of our ZIP code graph modeling are : (1) *ZIP Code Node* and (2) *IsAdjacentTo Edge*. A *ZIP Code Node* in Figure 6a maintains the basic information and an *IsAdjacentTo Edge* in Figure 6b connects two *ZIP Code Node*s with the derived information.

For the rest of this paper, we assume that the ZIP code graph that we use for measuring proximity is modeled by undirected graph *G*, where $V_{G}$ denotes the set of vertices (nodes) and $E_{G}$ represents the set of edges (adjacency). Table 4 shows the most frequently used symbols.

### 4.2. Graph Data Preprocessing

On the basis of the graph data modeling, we transform the raw data into graph data. Firstly, we discuss the processing of the ZIP code boundary dataset until the ZIP code adjacency is found. Then, we find the intersecting roads in the pair of adjacent ZIP codes and calculate the weight by utilizing a spatial index.

Details of building a ZIP code graph from ZIP code and a list of polygons are presented in Algorithm 1. In essence, there are five steps. In the first step, the algorithm creates the ZIP code graph by first iterating over all ZIP code details. In the second step, for each polygon, we create the polygon node and dissect it into its boundary points. We insert each point into graph databases and add the appropriate edges. In the third step, we execute another iteration, and, for each polygon, we identify the shared boundary points with other polygons and compute their lengths. We add the length into IsPolyAdjacent edges.

In the fourth step, for each ZIP code, we identify the polygon(s). Based on the IsPolyAdjacent edges, we insert the IsAdjacentTo edges and its properties by finding which ZIP code the other polygon belongs to and aggregate the common boundary length if needed. Finally, we delete all newly added edges and nodes because we do not need them anymore.
**Algorithm 1:** Build a ZIP codes Graph.
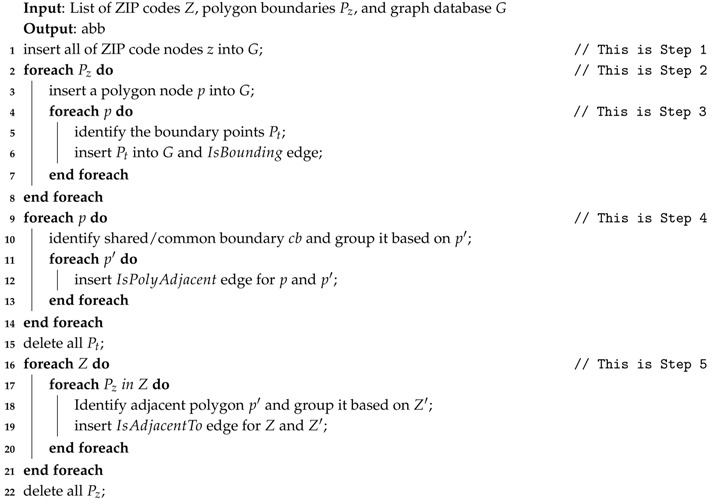

**Figure 7:**
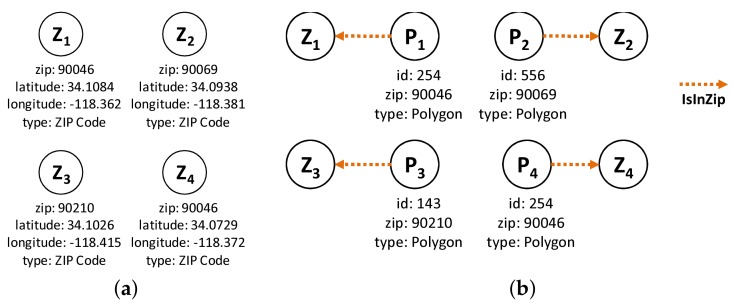
Building ZIP codes—Steps 1 and 2.1. (**a**) Step 1; (**b**) Step 2.1.

**Example** **2.** *On the basis of Figure 4a, we have four ZIP codes and four polygons with many boundary points for each polygon. Assuming that our ZIP code data are derived from Figure 4a. In the first step, we insert all four ZIP codes in G,* Step 1 *as shown in Figure 7a. Then, for each ZIP codes, we insert all the polygons and define an appropriate relationship between the ZIP code and the polygon.* Step 2.1 *in Figure 7b illustrates this process.*

**Figure 8:**
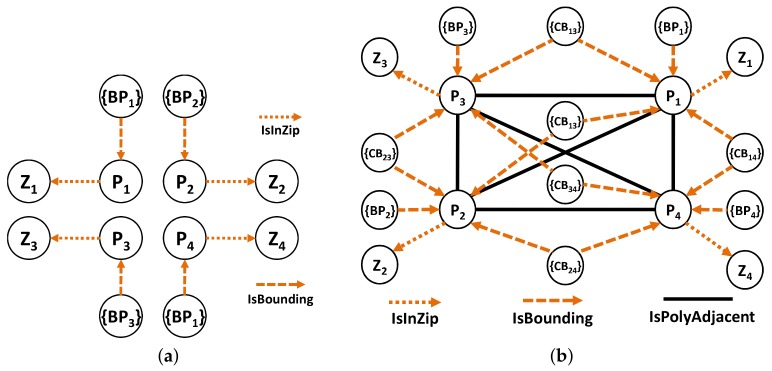
Building ZIP codes—Steps 2.2 and 3. (**a**) Step 2.2; (**b**) Step 3.

*After that, we identify the boundary points of each polygon and insert the appropriate relationships, which is the* Step 2.2 *in Figure 8a. Then, we identify the entire common boundary for each polygon by traversing over its boundary points and searching other polygons that used the same point and define the IsPolyAdjacent relationship. This step is illustrated as the* Step 3 *in Figure 8b.*

**Figure 9:**
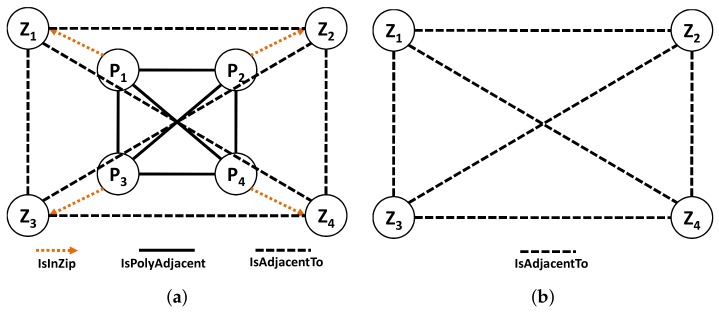
Building ZIP codes—Steps 4 and 5. (**a**) Step 4; (**b**) Step 5.

*After completely searching the common boundary and polygon adjacency, we delete all the boundary points and insert the IsAdjacentTo relationshi, which is* Step 4 *in Figure 9a. Finally, we delete all the polygon nodes and only maintain the ZIP code nodes and its adjacent relationship in* Step 5 *shown in Figure 9b.*

For finding the intersecting roads, we also employ the R-Tree index [25] as described in Algorithm 2. We build the R-Tree index for all of the TIGER/Line roads data and ZIP code boundaries. For each ZIP code, we find the polygon and iterate through its adjacent relationship to another ZIP code. Once we identify the polygons for adjacent ZIP codes, the computations for finding intersecting roads is executed. We assign the weight for each road type and update the adjacency relationship in the graph database storage. 

**Algorithm 2:** Finding Intersecting Roads.
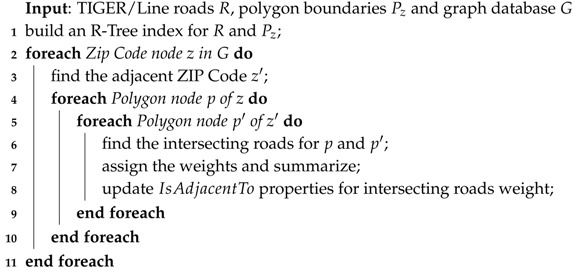

**Figure 10:**
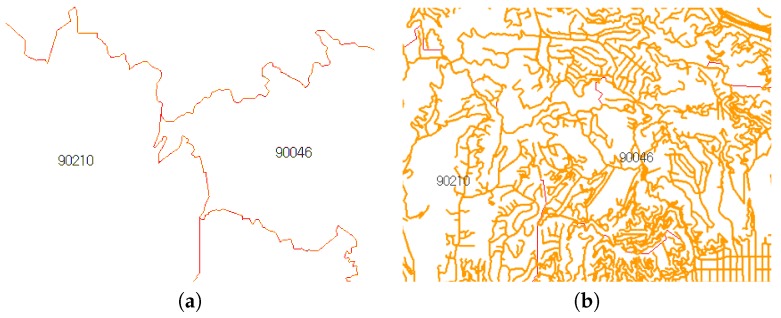
Roads in adjacent areas of 90210 and 90046. (**a**) plain adjacent ZIP code; (**b**) adjacent ZIP code with road network.

**Example** **3.** *The result of Algorithm 1 is a ZIP code node and its adjacency. Based on this adjacency, we try to find the intersecting roads between the polygons of adjacent ZIP codes. First, we build an R-Tree index for both the ZIP code polygons and the TIGER/Line data. As shown in Figure 10a, ZIP 90046 is adjacent to 90210. We found that there are 15 roads that intersect between their polygons as illustrated in Figure 10b. Among these 15 roads, there are no primary roads, one secondary road and 14 other roads. Using Definition 3, we calculate the intersecting roads weight of 90046 (s) and 90210 (t) as follows:*

$$\begin{array}{cc} rw_{\left(s,t\right)}\; & =\left(n_{s1100}\right)\left(w_{s1100}\right)+\left(n_{s1200}\right)\left(w_{s1200}\right)+\left(n_{s1400}\right)\left(w_{s1400}\right)\\ & =(0)(1)+(1)(0.5)+(14)(0.2)\\ & =(0)(1.0)+(1)(0.5)+(14)(0.2)\\ & =1.9. \end{array}$$

## 5. Efficient Proximity Computation

In this section, we describe the processing details of the proximity query engine module. Firstly, we explain the proximity measures that we use, including the reasoning and the mathematical equations that conform to the distance measure properties. Then, we describe the basic proximity computation of the adjacent and non-adjacent ZIP codes. For non-adjacent codes, we further divide the computation into *Ad-Hoc* and *Top-K* computation. Lastly, we propose a heuristic approach for efficient proximity computation by trying to reduce the required search space by using the past and future distances.

### 5.1. Proximity Measures

Based on the Definitions 2 and 3, it is emphasized that our proximity value should always minimize distance while maximizing the intersecting roads weight. In [4], the research discusses a similar situation with us. They use two distance measurements for computing proximity, which are the shortest path and the modified maximum flow. To combine these two methods, the researchers use the product of the shortest path and the maximum flow. To obtain the optimum value, both measurements must be minimized. However, the result of product combination does not conform to the triangle inequality; thus, it is not a distance measurement.

A distance measurement should follow the distance metric properties: (1) d(x,y)≥0, *non-negativity*; (2) d(x,y)=0, if and only if x=y *identity of indiscernibles*; (3) d(x,y)=d(y,x), *symmetry*; and (4) d(x,z)≤d(x,y)+d(y,z), *triangle inequality*. If our new function follows this metric, we can guarantee that the inversely related distance with proximity will hold true.

For the purpose of making the new proximity value conform to the distance metrics, we model the problem definition as a multi-objective optimization with preferences for a relatively short centroid distance. In single-objective optimization, there is always a single global solution. However, in multi-objective optimization, typically, there is no single global solution. It is necessary to have a preference for an objective function that has a relatively high priority [26].

Our objective function consists of two different targets. First and foremost, we must convert all objective functions into the same target function, either minimize all or maximize all. The current centroid distance is already a minimize function, but the intersecting roads weight is not:

(1) $$min\left(dist_{\left(v_{s},v_{t}\right)}\right)=min\left(\sum_{i=1}^{k}dist_{\left(v_{i},v_{i+1}\right)}\right).$$

We convert the intersecting roads weight into a minimizing function by subtracting its maximum value from the current value:

(2) $$max\left(rw_{\left(v_{s},v_{t}\right)}\right)=max\left(\sum_{i=1}^{k}rw_{\left(v_{i},v_{i+1}\right)}\right)\equiv min\left(\sum_{i=1}^{k}\left(\left\lceil MAX_{rw}\right\rceil -rw_{\left(v_{i},v_{i+1}\right)}\right)\right).$$

With respect to the pair of ZIP codes, the maximum value of the intersecting roads weight is as follows:

(3) $$\begin{array}{c} MAX_{rw}=\left\{\begin{array}{cc} 0, & \mathrm{if}\;i=(i+1),\\ MAX_{rw}, & \mathrm{if}\;i\neq (i+1). \end{array}\right. \end{array}$$

As the following lemma shows, the new minimize function of common boundary respects all distance metric properties.

**Lemma** **1.** The new minimize function of the intersecting roads weight as defined in Equation (2) respects the distance metric properties (non-negativity, identity of indiscernibles, symmetry, and triangular inequality).

**Proof.**  Let *G* be an undirected graph and u,v be two nodes of *G*. It is evident that, if u≡v, the intersecting roads weight is 0, which satisfies *identity of indiscernibles*. If u≠v, the intersecting roads weight is non-zero, thus satisfying the condition of *non-negativity*. Since *G* is an undirected graph, the intersecting roads weight from *u* to *v* is equal to the intersecting roads weight from *v* to *u*, and thus, *symmetry* is also satisfied.

Let us prove that *triangular inequality* also holds. Let p(v,u) be the unique path connecting *u* and *v*. Furthermore, *x* belongs to the path *p*, as in *x* is located between *u* and *v*. On the basis of triangular inequality, our intersecting roads weight function should satisfy

$$MAX_{\left(rw\right)}-rw_{\left(u,v\right)}\leq MAX_{rw}-rw_{\left(u,x\right)}+MAX_{rw}-rw_{\left(x,v\right)}.$$

Assuming that $MAX_{rw}=m_{c}$, $rw_{\left(u,x\right)}=c_{1}$, and $rw_{\left(x,v\right)}=c_{2}$, we can prove that the *triangle inequality* holds as follows:

$$\begin{array}{cc} MAX_{rw}-rw_{\left(u,v\right)} & \leq MAX_{rw}-rw_{\left(u,x\right)}+MAX_{rw}-rw_{\left(x,v\right)}\\ MAX_{rw}-\left(rw_{\left(u,x\right)}+rw_{\left(x,v\right)}\right) & \leq MAX_{rw}-rw_{\left(u,x\right)}+MAX_{rw}-rw_{\left(x,v\right)}\\ m_{c}-c_{1}+c_{2} & \leq m_{c}-c_{1}+m_{c}-c_{2}\\ m_{c}-c_{1}+c_{2} & \leq 2m_{c}-c_{1}+c_{2}. \end{array}$$

 ☐

We use the most common approach to solve the problem of combining both the centroid distance and the common boundary, namely the *Weighted Sum Method*. By applying this method to our proximity computation, based on Equation (1) for centroid distance and Equation (2) for the intersecting roads weight, we obtain the new proximity equation as follows:

(4) $$\begin{array}{cc} prox(v_{s},v_{t})\; & =\left(\alpha \right)\left(min\left(dist_{\left(v_{s},v_{t}\right)}\right)\right)+\left(1-\alpha \right)(max\left(rw_{\left(v_{s},v_{t}\right)}\right), \end{array}$$

(5) $$\begin{array}{cc} prox(v_{s},v_{t})\; & =\left(\sum_{i=1}^{k}\left(\alpha \left(dist_{\left(i,i+1\right)}\right)+\left(1-\alpha \right)\left(\left\lceil MAX_{rw}\right\rceil -rw_{\left(i,i+1\right)}\right)\right)\right). \end{array}$$

For adjacent ZIP codes, we can simplify Equation (4). Assuming that $v_{s}$ and $v_{t}$ is adjacent, we can express the proximity computation as follows:

(6) $$prox\left(v_{s},v_{t}\right)=\left(\alpha \left(dist_{\left(v_{s},v_{t}\right)}\right)+\left(1-\alpha \right)\left(MAX_{rw}-rw_{\left(v_{s},v_{t}\right)}\right)\right).$$

If we choose to compute the proximity for adjacent ZIP codes in the preprocessing stage, we can use it to compute the proximity for non-adjacent ZIP codes. Assuming that $v_{i}$ and $v_{i+1}$ is adjacent, we can express the equation as follows:

(7) $$\begin{array}{c} w_{\left(v_{i},v_{i+1}\right)}=prox_{\left(v_{i},v_{i+1}\right),} \end{array}$$

(8) $$\begin{array}{c} prox\left(v_{s},v_{t}\right)=min\left(\sum_{i=1}^{k}w_{\left(v_{i},v_{i+1}\right)}\right). \end{array}$$

As the following lemma shows, this proximity function also respects all distance metric properties.

**Lemma** **2.** The proximity function $prox(v_{s},v_{t})$ as defined in Equation (4) respects the distance metric properties (non-negativity, identity of indiscernibles, symmetry, and triangular inequality)

**Proof.**  If two or more distance measures respect the distance metric properties, then so does any linear combination of these measures. The centroid distance already satisfies the distance metric properties, and so does the new intersecting roads weight function, which has been proven in Lemma 1. ☐

**Figure 11:**
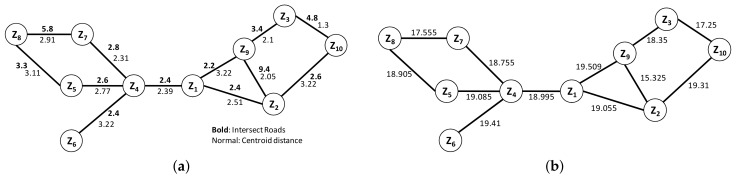
Proximity computation. (**a**) Zip code graph example. (**b**) ZIP code graph with adjacent proximity.

**Example** **4.** *The partial result of building a graph from ZIP code data is shown in Figure 11(a), where weights in bold font denote the intersecting roads weight, those in normal font represent the centroid distance and $MAX_{\left(rw\right)}=37.5$. We can compute the proximity of adjacent ZIP code by using Equation (6). For example, between $Z_{1}$ and $Z_{4}$ with alpha=0.5, $rw_{\left(z_{1},z_{4}\right)}=2.4$ and $dist_{\left(z_{1},z_{4}\right)}=2.39$:*

$$\begin{array}{cc} prox(v_{s},v_{t})\; & =\left(\alpha \left(dist_{\left(v_{s},v_{t}\right)}\right)+\left(1-\alpha \right)\left(MAX_{rw}-rw_{\left(v_{s},v_{t}\right)}\right)\right)\\ & =(0.5)(2.39)+(1-0.5)(37.5-2.4)\\ \; & =18.995. \end{array}$$

We calculate the other adjacent ZIP code proximity by using the same process and we obtain the result as shown in Figure 11b.

### 5.2. Non-Adjacent (Ad-Hoc and Top-K) Proximity Processing

As we can see in Algorithm 3, based on the user input, we can divide the proximity computation into two parts. The first one measures the *Ad-Hoc* proximity and provides a GraphML file and a proximity value. The other one measures the *Top-K* proximity or the node’s neighborhood and provides a GraphML file for the topology and *k*-zip codes with the proximity value.
**Algorithm 3:** Proximity Computation.
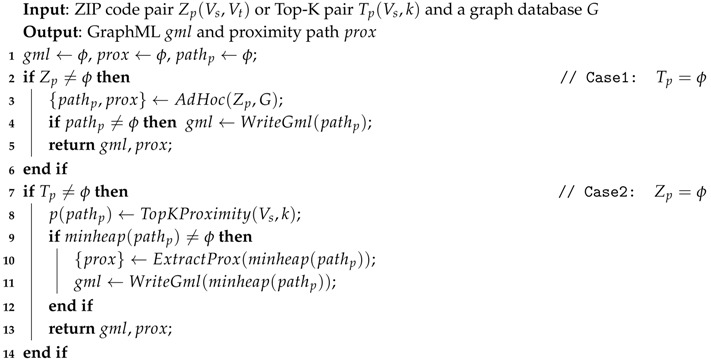

**Example** **5.** We will use graphs from Figure 11b for the rest of the example. Supposed that we want to find the proximity between $Z_{1}$ and $Z_{10}$. Based on Equation (3), this computation can be categorized as Ad-Hoc proximity computation and further processed using Algorithm 4. If there is a path connecting them, we will extract the proximity value from the path and write into a GraphML file. In other example, if we want to find the top five neighborhood proximities of $Z_{1}$, the computation will be categorized as Top-K proximity computation and Algorithm 5 will be used for computing it. The result of this computation is a minheap that consists of the path and the proximity value.

We divide the *Ad-Hoc* proximity computation into two parts: (1) If the *source* and *target* ZIP codes are adjacent, we can directly compute the proximity using adjacency properties: centroid distance and intersecting roads weight. (2) If the *source* and *target* ZIP codes are not adjacent, then we need to find the minimum sum of the centroid distance and the intersecting roads weight using Equation (4). Algorithm 4 shows details of our *Ad-Hoc* computation.
**Algorithm 4:** Ad-Hoc Proximity Computation.
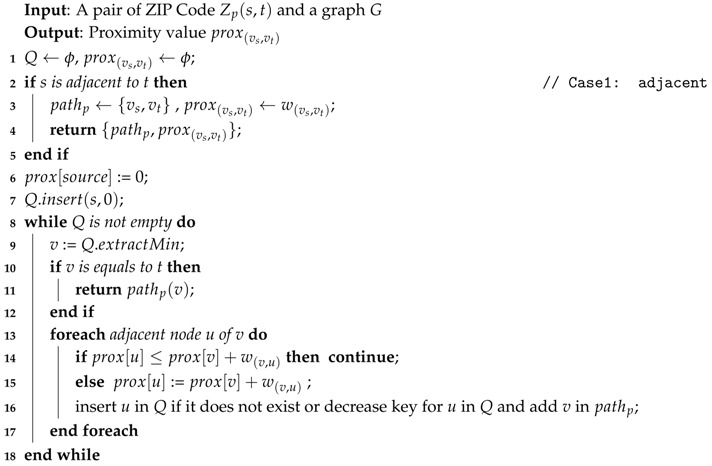

**Example** **6.** Let us continue from Example 5 and process $AdHoc\left(z_{1},z_{10}\right)$ using Algorithm 4. First, we define the proximity of $Z_{1}=0$. Then, we traverse and count the weight of the adjacent nodes of $Z_{1}$, which are $Z_{9}$,$Z_{2}$, and $Z_{4}$. This process is continued until we get to the node $Z_{10}$ and return the result, path and proximity value. In this case, the $path=\{Z_{1},Z_{2},Z_{10}\}$ with $prox_{\left(z_{1},z_{10}\right)}=38.365$.

To compute the node’s neighborhood or *Top-K* proximity, we use the *MinMaxHeap* to store the current findings of the node-*k* and its sorted proximity value. While the size of the heap is still less than *k*, we keep inserting the node pair. However, if the heap is already full and the next value of proximity is no better than the worst value in the heap, we consider the computation complete and return the result.
**Algorithm 5:** Top-K Proximity Computation.
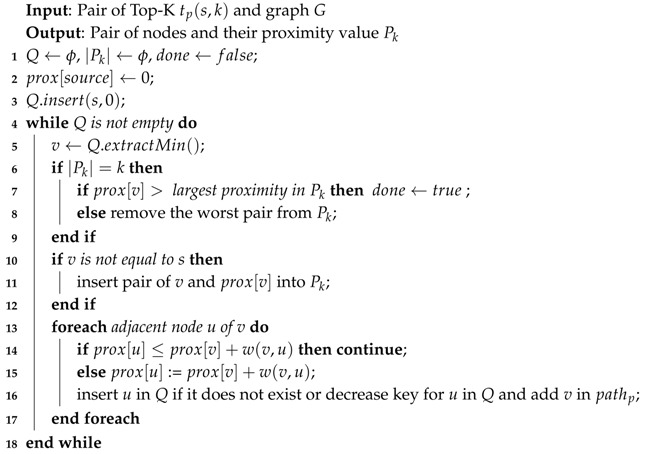

**Example** **7.** Let us continue from Example 5 and process $Top-K\left(z_{1},5\right)$ using Algorithm 5. First, we initialize all variables and define the proximity of $Z_{1}=0$. Then, we traverse and count the weight of the adjacent nodes of $Z_{1}$, which are $Z_{9}$,$Z_{2}$, and $Z_{4}$. In each iteration, after we extract the minimum value from the priority queue, we insert it into $P_{k}$. We continue this process this way until we obtain the k node(s) in $P_{k}$.

### 5.3. Heuristic Proximity Computation

**Figure 12:**
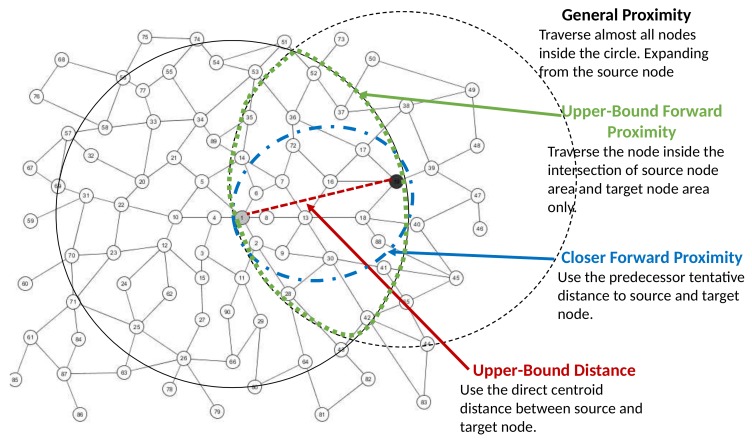
Search space difference.

We have to minimize the search space if we want to decrease the running time. Although the complexity remains the same, but the running time can be decreased. The idea here is to use the *past-path distance* to keep the proximity search always forward. We add more constraints in the algorithm. For instance, the past-path distance of the next traversed node or node in the *priority queue* *Q* should always be bigger or equal to that of the current node. Furthermore, we add more constraints for *past-path distance* and *future-path distance* to be within the upper bound of the direct centroid distance from $V_{s}$ to $V_{t}$. However, this approach can only be used for *Ad-Hoc* proximity computation. We call this approach as *Upper-Bound Forward* approach. Algorithm 6 shows our *Upper-Bound Forward*. The search space difference of this approach is illustrated in Figure 12.

We can minimize the search space further by tightening the constraint of *future-path distance*. As in the case of *Upper-Bound Forward*, the complexity remains the same but with reduced running time. If no path is found, we will relax the constraint as in the case of *Upper-Bound Forward*. Here, we enforce that the future-path distance of the next traversed node be smaller or equal to the current node or its predecessor. We call this approach *Closer Forward* and use Algorithm 7 to explain it.

The *Upper-Bound Forward* and *Closer Forward* approaches can only be used for estimating *Ad-Hoc* proximity. We cannot use them from computing the *Top-K* proximity. We can only use the *Forward* approach with *MinMaxHeap* to store the result of the shortest path computation. The parameter *k* is used for defining the size of *MinMaxHeap*. When the heap is in its full size, our computation stops because there are no more better solutions to be found.
**Algorithm 6:** Upper-Bound Proximity.
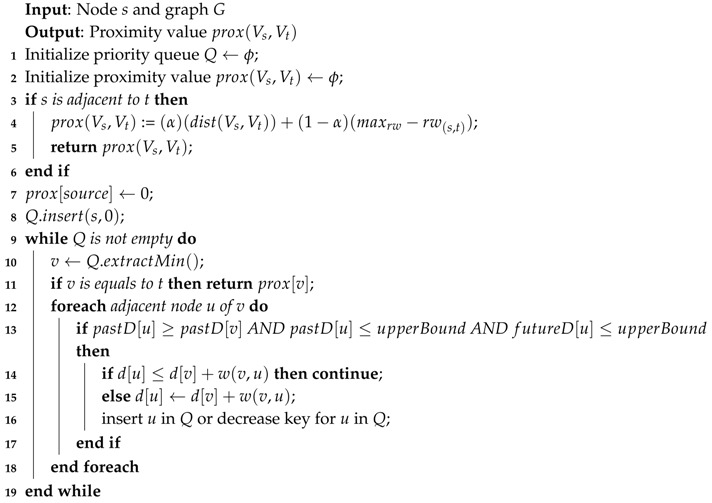

**Algorithm 7:** Closer Forward Proximity.
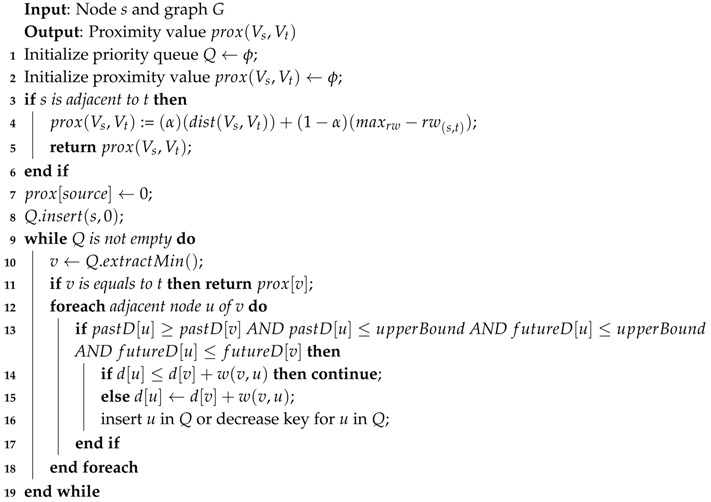

**Figure 13:**
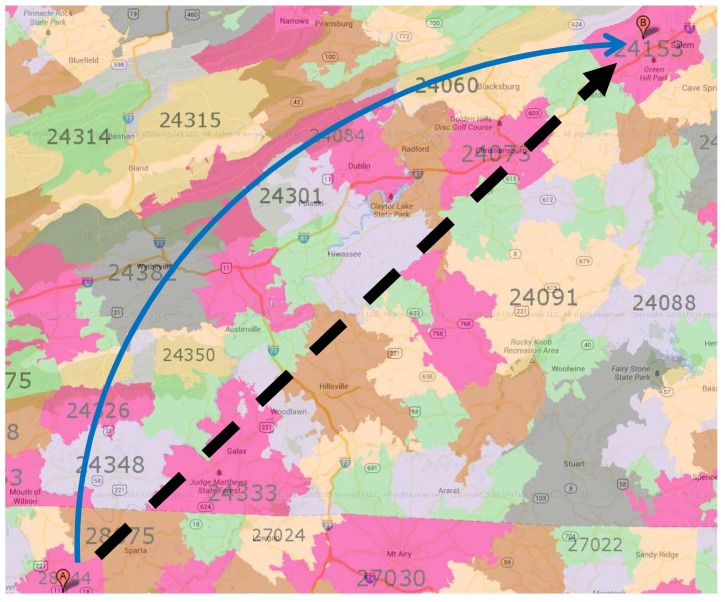
Example of heuristic proximity computation.

**Example** **8.** *As shown in Figure 13, we want to find the proximities from 28644 to 24153. If we use the general approach, the number of traversed nodes is 462, since the general approach traverses almost all nodes inside the circle of the source and target node distance. The* Upper-Bound Forward *method only traverses nodes in the forward direction, and the number of traversed nodes reduces to 186, which is almost half of that obtained by using the general method. The* Closer Forward *approach reduces the number of traversed nodes significantly to only 84 nodes, since it only finds the optimum distance that is closer to or equal to the current distance.*

### 5.4. Case Studies for Zip Code Proximity Computation

We will justify the correctness of our proximity computation by using four different sets of ZIP codes example and provide the result of the graph building proximity computation.

**Figure 14:**
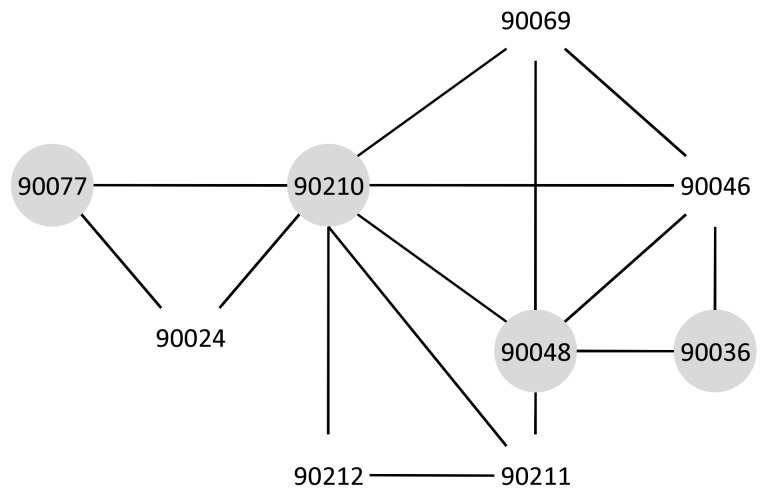
General ZIP code graph.

**Example** **9.** (General ZIP Codes) There is no barrier within this set of ZIP codes as is shown in Figure 14. This figure shows the general case for ZIP codes, e.g., the proximity between 90077 and 90036. Figure 14 shows the graph of a general ZIP codes set. It shows the result of the Ad-Hoc proximity computation as the proximity path from 90077 to 90036 in the gray area: 90077–90210–90048–90036. Since there are no barriers, the graph building and proximity computation is a rather simple task.

**Figure 15:**
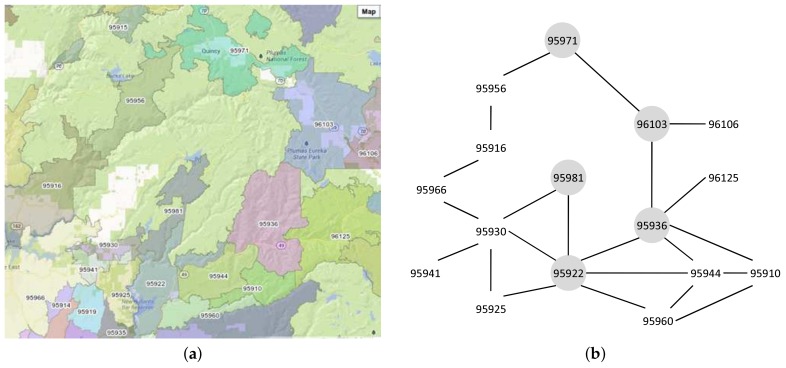
Mountain-separated ZIP code graph. (**a**) map; (**b**) ZIP code graph.

**Example** **10.** (ZIP codes separated by mountain) In this set of ZIP codes, there is an impassable mountain that separates two ZIP codes. Figure 15a shows this ZIP code set. It shows that, between 95981 and 95971, there is an impassable wide mountain. Thus, we need to take a detour to find the proximity path.

Figure 15b shows the graph of mountain-separated ZIP codes set. The result of the Ad-Hoc proximity computation is illustrated in the gray areas. The proximity path from 95971 to 95981 is 95971–96103–95936–95922–95981. Looking at the map, we find that the centroid distance for 95971 and 95981 is not large, but, because there is a mountain between these ZIP codes, we have to take a detour. The detour is shown by the result of our proximity computation. Thus, the correctness of the proximity computation is verified.

**Figure 16:**
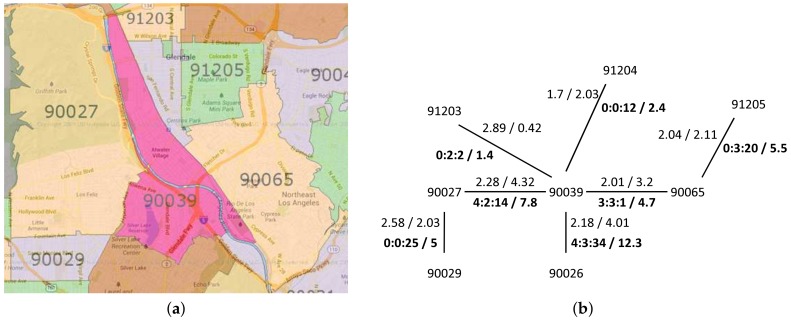
River-separated ZIP code graph. (**a**) map; (**b**) ZIP code graph.

**Example** **11.** (ZIP codes separated by a river) In this set of ZIP codes, there is a passable river that separates two ZIP codes. Although they share lengthy common boundaries, they are only connected by several roads. An example of this set is 90039 and 90027, shown in Figure 16a.

Figure 16b shows the graph of the river-separated ZIP codes set. There are two sets of weights value for each adjacent relationship. The normal type shows the set of the centroid distance and the common boundary length, while the bold type shows the set of the intersecting roads in the form of (primary::secondary::other) and the sum of the assigned road weights.

As we can see in Figure 16a, Los Angeles River runs alongside the boundary of 90039 and 90027. Because of this river, although the common boundary length value is high, the number of intersecting roads is not large. For the sake of comparison, let us look at 90039 and 90026. Their common boundary length is smaller, but they have higher intersecting roads since there are no rivers alongside their boundaries. Thus, our intersecting roads measurement correctness is verified.

## 6. Experiments

In this section, we provide the details of the experimental environments and results.

### 6.1. Experimental Setup

#### 6.1.1. Environments

We ran our experiments on an Intel Core I5 2.67 GHz machine with 4 GB of memory running Linux Mint 14. Our algorithms and graph building were implemented in *JAVA* using JDK version 1.6. We used the current stable release of Neo4j [27], which is a community edition of *Neo4j Stable Release 1.9*.

#### 6.1.2. Datasets

The performance evaluation study is based on a real ZIP code boundary dataset [24], which consist of 33,174 ZIP codes with more than 39,000 polygons and 28 million boundary points. The intersecting TIGER/Line roads data are identified using PostGIS version 2.0 [28]. The data is available form the web site of the United States Census Bureau [20]. In total, there are 3209 shapefiles of roads covering all counties in a state, and the extracted size of the dataset is 4.4 GB.

Because of the considerable number of boundary points and the required computation to identify the common boundary length, our ZIP code graph building takes more than one and a half days. The storage needed for storing the resulted graph is almost 100 GB. However, later, after cleaning up the unnecessary nodes and relationships, the required storage becomes considerably smaller—only 200 MB. The final dataset contains 33,174 ZIP codes and around 178,000 adjacency relationships.

#### 6.1.3. Queries

To see the performance of three approaches in terms of *Ad-Hoc* proximity, we used the close pair and the far pair query types, which are based on the distance. Table 5 explains the characteristics of these query types.

To see the effect of natural barriers in the *Ad-Hoc* proximity computation, we used the three query types reflecting the natural barriers between two ZIP codes. Table 6 explains the details of these query types.

For testing *Top-K* proximity queries, we randomly selected ZIP codes and varied the value of *K*.

#### 6.1.4. Metric

For *Ad-Hoc* proximity, the metrics for verifying a successful experiment that we use are the number of traversed nodes and the running time of the proximity computation. For *Top-K* proximity, we show the execution time on the logarithmic scale and the memory usage of our algorithm. To make the experimental results more sound and reliable, we conducted the test in 10 times and averaged all the reported experimental results.

### 6.2. Experimental Results

As mentioned before, all experiments use the ZIP code graph and the proximity measure between nodes is expressed by a linear combination of the centroid distance and the intersecting roads weight, as shown in Equation (4).

#### 6.2.1. Varying Alpha

In this experiment, we varied the value of alpha, which is used as a weight for the centroid distance and the intersecting roads weight between two ZIP codes, and used the general case query type.

**Figure 17:**
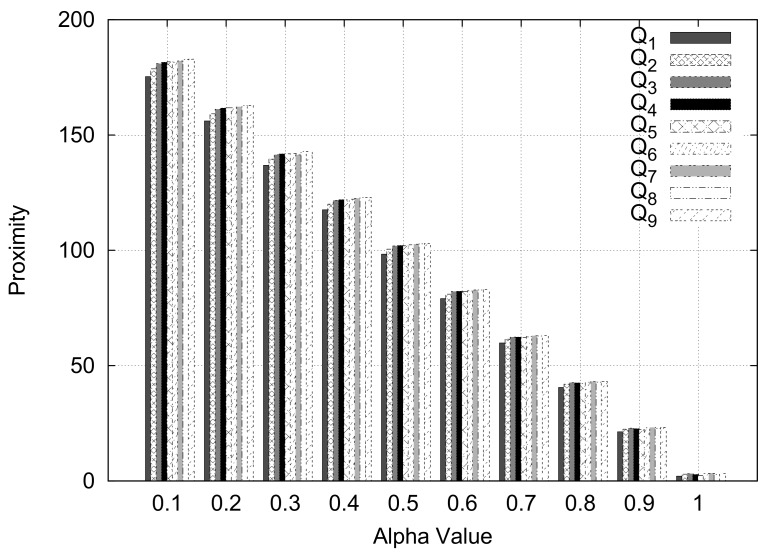
Varying value of alpha.

Figure 17 depicts the experimental results. We can see that the proximity values of ten queries are consistently preserved for the varying alpha (weight) values. From the experiment, we can conclude that the proximity results are well preserved although we assign different alpha values. Thus, we will choose 0.5 as a weight value for the centroid distance and the intersecting roads weight between two ZIP codes for the following experiments.

#### 6.2.2. *Ad-Hoc* Proximity Computation

In this subsection, we evaluate the performance of our approach for processing *Ad-Hoc* proximity queries.

#### 6.2.3. Using Distance-Based Query Types

In this experiment, we compare the running time and also the number of traversed nodes using the general, *Upper-Bound*, and *Closer Forward* methods.

**Figure 18:**
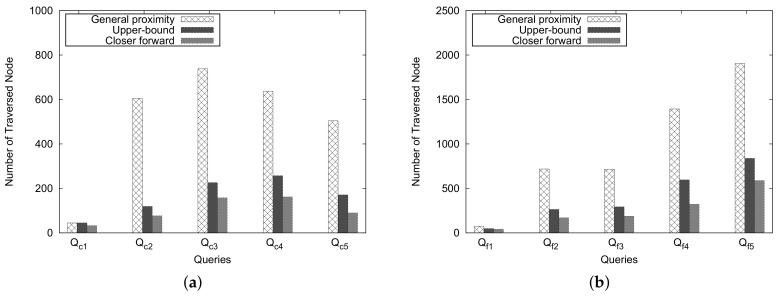
Number of traversed nodes for general cases. (**a**) close pair queries; (**b**) far pair queries.

Figure 18 shows the number of traversed nodes for these approaches. An important trend is observed from this figure: as the number of traversed nodes grows, the *Upper-Bound* and *Closer Forward* methods can reduce the number of traversed nodes dramatically compared with the general method for processing close pair/far pair zip codes. However, the number of traversed nodes differs slightly if the number of traversed nodes is less than 50 ( $Q_{c1}$ and $Q_{f1}$ in Figure 18a,b).

**Figure 19:**
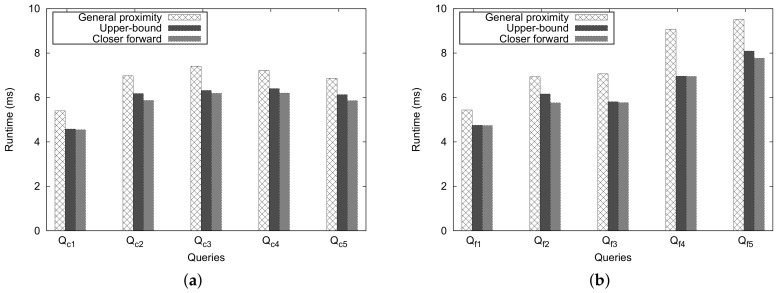
Execution time for general cases. (**a**) close pair queries; (**b**) far pair queries.

Figure 19 shows the execution time for the *Ad-Hoc* proximity computation. An important observation is that, as we expected, the *Closer Forward* method shows better performance than the *Upper-Bound* and the general approaches, and the performance of *Upper-Bound* method is considerably better than that of the general approach. Another observation is that, even when we face some extreme cases of ZIP code pairs such as $Q_{c1}$ and $Q_{f1}$, then the running time and the number of traversed nodes differ only slightly. This is attributed to the overhead of checking the heuristic estimate; however, for the general proximity, there is no overhead.

#### 6.2.4. Using Natural-Barrier Query Types

In this experiment, we randomly selected ZIP code pairs by considering the natural barrier between two ZIP codes. To compare the runtime computation and the number of traversed nodes, we executed queries for five pairs, ranging from close pair to far pair, for each natural barrier.

**Figure 20:**
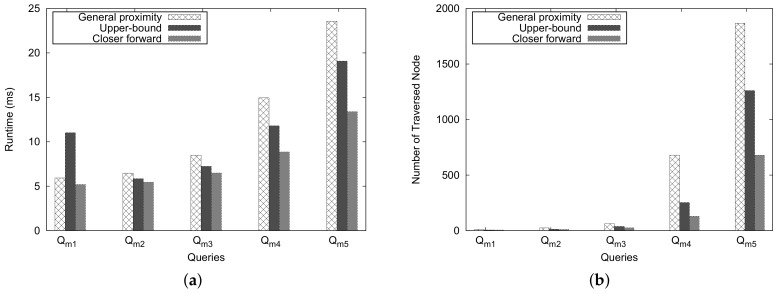
Mountain-separated case. (**a**) execution time; (**b**) number of traversed nodes.

Figure 20 depicts the experimental results for the mountain-separated case. The general approach costs considerably more than *Upper-Bound* and *Closer Forward* approaches in most pairs of queries. However, only for a query such as ($Q_{m1}$), the *Upper-Bound* method shows worse performance than the general and *Closer Forward* approaches. This is due to the fact that when the number of traversed nodes is almost the same, the overhead for checking the heuristic estimate of the *Upper-Bound* approach affects the execution time.

Let us analyze the performance by comparing the results of $Q_{m1}$ and $Q_{m5}$. When we see the number of traversed nodes in Figure 20b, the number of traversed nodes for $Q_{m5}$ is 20 times more than that for $Q_{m1}$. However, the execution time of $Q_{m5}$ as shown in Figure 20a is only four times more than that of $Q_{m1}$ in the case of the general approach.

**Figure 21:**
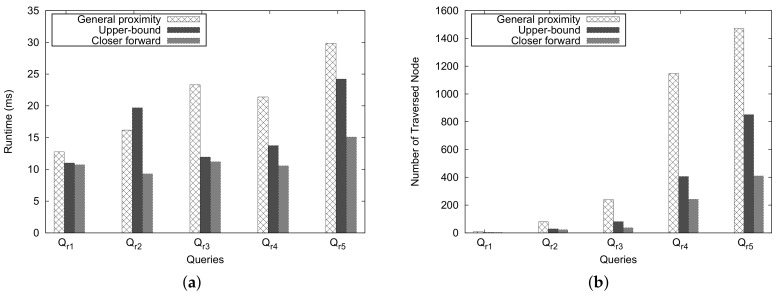
River-separated case. (**a**) execution time; (**b**) number of traversed nodes.

Figure 21 depicts the experimental results for the river-separated case. Similar to the mountain-separate case, the general approach costs considerably more than *Upper-Bound* and *Closer Forward* approaches in most pairs of queries. However, only for a query such as ($Q_{r2}$), the *Upper-Bound* method shows worse performance than the general and *Closer Forward* approaches.

According to the experiment results, we can say that our heuristic approach can reduce the number traversed nodes significantly and effectively reduce the execution time in most cases.

#### 6.2.5. *Top-K* Proximity Computation

In this experiment, we analyze the execution time and the memory usage for the *Top-K* proximity queries.

**Figure 22:**
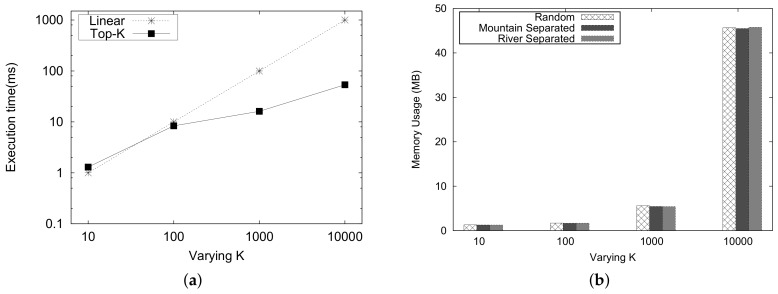
*Top-K* proximity computation. (**a**) execution time; (**b**) memory usage.

Figure 22a shows the runtime of *Top-K* for *k* on the logarithmic scale. For the *y* scale, the runtime is represented on the logarithmic scale of 10. The computation runtime of *Top-K* proximity is considerably better than the linear processing time as the *k* increases.

The memory usage for the *Top-K* proximity queries is depicted in Figure 22b. As the values of K increases, the memory usage also increases. The memory usage for different types of queries is almost the same.

## 7. Conclusions

In this paper, we have designed and evaluated an efficient proximity computation system using ZIP code graph data for smart city applications. For this purpose, we first define a novel way to measure proximity using intersecting road networks and the centroid distance of the adjacent node along the path. We also provide a mathematical model and use the weighted sum for combining intersecting road networks and the centroid distance. Next, we propose efficient proximity computation methods such as *Upper-Bound* and *Closer Forward* approaches.

Our system can handle two types of proximity computations, namely the *Ad-Hoc* and *Top-K* proximity. For *Ad-Hoc*, we need the ZIP code pair input from the user and the result is the proximity value. In contrast, for *Top-K*, we need the source ZIP code and the value of *k*. The output are the *k* zip codes with their own proximity value. We use a priority queue and MinMaxHeap for computing the proximity value. As was clearly demonstrated by the experimental results, our system can exhibit good performance for *Ad-Hoc* and *Top-K* proximity queries in ZIP codes graph data.

We plan to extend our work in several directions. We would like to develop a graph partitioning technique for more efficient proximity computation. Since our current work focuses on only road networks, we plan to generalize the proposed techniques and apply them to the graph data of a social network. Intuitively, while transforming social network data into a graph database, we would like to consider direct/indirect relationships as distance and the number of tweets or posts as the common boundary points between two people.

## Figures and Tables

**Table 1:** Research on proximity computation in a graph.

Research	Proximity Measurement	Dataset Type
Person search [8], link prediction [9,10,11]	Common neighborhood number	Social network graph
RWR (Random Walk with Restart) [5]	Steady-state probability	Image
TAGs (Threshold Algorithms for Graphs) [4]	Shortest path distance and the maximal network flow	Gene Expression, Web links, road network, etc.
Our work	Intersecting road weight between adjacent ZIP codes	Road network

**Table 2:** Research on top-k spatial queries.

Research	Key Features
Yiu et al. [14]	Set of quality features (non-spatial) of spatial neighborhood
Tsatsanifos et al. [7]	Set of Spatio-Textual Preference features
Zheng et al. [6]	Feedback-based Spatial-keywords Preference features
ALPS (ALgorithm for top-k spatial Preference Search) [16]	Skyline set from transformed data objects in road segments
COMET (COllaborative approach to Moving k nEaresT neighbor) [17]	k-NN on moving query object in directed and dynamic road networks
Luo et al. [18]	Reverse spatial and textual k-Nearest Neighbor (RSTkNN) on road networks
Our initial work [19]	Common boundaries features and proximity features based on centroid distance
Our work	Proximity features based on intersecting road weight between adjacent ZIP codes

**Table 3:** Research on a minimum distance computation algorithm.

Research	Key Features
Breadth-First Search [21]	Implementation of queue for unweighted graph, expensive for graph with high degree of connectivity
Dijkstra [22]	Non-negative valued weighted graph minimum distance search
A* search [23]	Greedy approach of minimum distance search with heuristic value
Our work	A* search based with increase of degree of relaxation

**Table 4:** Frequently used symbols.

Symbol	Definition
*G*	an undirected and weighted graph
$v_{i}$	the *i*-th node of a graph
*k*	number of nodes in the path connecting two nodes
$w_{\left(v_{s},v_{t}\right)}$	weight of the edge between node $v_{s}$ and $v_{t}$
$dist_{\left(v_{s},v_{t}\right)}$	centroid distance between node $v_{s}$ and $v_{t}$ in *G*
$min(dist_{\left(v_{s},v_{t}\right)})$	minimum centroid distance summation for adjacent node in a path between $v_{s}$ and $v_{t}$ in *G*
$rw_{\left(v_{s},v_{t}\right)}$	intersecting roads weight between node $v_{s}$ and $v_{t}$ in *G*
$max(rw_{\left(v_{s},v_{t}\right)})$	maximum intersecting roads weight summation for adjacent node in a path between $v_{s}$ and $v_{t}$ in *G*
$MAX_{rw}$	maximum value of intersecting road weights in *G*
α	weight value preference for centroid distance
$prox_{\left(v_{s},v_{t}\right)}$	proximity value between $v_{s}$ and $v_{t}$ in *G*

**Table 5:** Query types based on distance.

Query Type/Notation	Description
ClosePair/$Q_{c}$	This query consists of two ZIP Codes (source and target), which the number of traverse node is less than thousands, and the centroid distance between two nodes not larger than 150.
FarPair/$Q_{f}$	This query consists of two ZIP Codes (source and target), which the number of traverse nodes starts from 500 (for general approach) and the distance between them is more than 60.

**Table 6:** Query types based on the natural barriers.

Query Type/Notation	Description
General/$Q_{g}$	This query consists of two ZIP Codes (source and target) without the natural barriers between them.
MountainSeparated/$Q_{m}$	This query consists of two ZIP Codes (source and target) that are separated by mountains.
RiverSeparated/$Q_{r}$	This query consists of two ZIP Codes (source and target) that are separated by rivers.
